# Chromosome-specific oligo-painting provides insights into the cytogenetic basis of karyotypic stasis in paleo-allotetraploid *Cucurbita*

**DOI:** 10.1093/hr/uhaf179

**Published:** 2025-07-08

**Authors:** Qinzheng Zhao, Yulin Bai, Yuhui Wang, Chunyan Cheng, Xiaqing Yu, Qunfeng Lou, Jinfeng Chen

**Affiliations:** State Key Laboratory of Crop Genetics & Germplasm Enhancement and Utilization, College of Horticulture, Nanjing Agricultural University, 1 Weigang, Nanjing, Jiangsu 210095, China; State Key Laboratory of Crop Genetics & Germplasm Enhancement and Utilization, College of Horticulture, Nanjing Agricultural University, 1 Weigang, Nanjing, Jiangsu 210095, China; State Key Laboratory of Crop Genetics & Germplasm Enhancement and Utilization, College of Horticulture, Nanjing Agricultural University, 1 Weigang, Nanjing, Jiangsu 210095, China; State Key Laboratory of Crop Genetics & Germplasm Enhancement and Utilization, College of Horticulture, Nanjing Agricultural University, 1 Weigang, Nanjing, Jiangsu 210095, China; State Key Laboratory of Crop Genetics & Germplasm Enhancement and Utilization, College of Horticulture, Nanjing Agricultural University, 1 Weigang, Nanjing, Jiangsu 210095, China; State Key Laboratory of Crop Genetics & Germplasm Enhancement and Utilization, College of Horticulture, Nanjing Agricultural University, 1 Weigang, Nanjing, Jiangsu 210095, China; State Key Laboratory of Crop Genetics & Germplasm Enhancement and Utilization, College of Horticulture, Nanjing Agricultural University, 1 Weigang, Nanjing, Jiangsu 210095, China

## Abstract

Post-polyploid karyotype evolution represents a crucial cytological mechanism contributing to angiosperm diversification and speciation. Many polyploids show extensive karyotypic reshuffling relative to their pre-ancestors. However, karyotypic stasis is gaining popularity as an alternative evolutionary pathway following polyploidization, whose underlying cytological mechanisms remain poorly understood. Here, we successfully developed a set of enhanced oligo-painting (EOP) probes specific to 20 chromosomes of *Cucurbita* (2*n* = 40), a paleo-polyploid with very small chromosomes and rich genetic diversity. The probes generated robust fluorescence *in situ* hybridization (FISH) signals across six *Cucurbita* and one sister outgroup species. Cross-species EOP results confirmed that *Cucurbita* genomes originated from a paleo-allotetraploid and maintained remarkably conserved chromosomal synteny without chromosome reshuffling, indicating karyotypic structural stasis during post-polyploid diploidization. Repositioning and amplification/elimination of rDNA loci (45S and 5S) across species caused significant morphological variations on seven out of 20 chromosomes. Six predicted centromeric monomers showed dramatic variations in localization and copy number along the phylogenetic relationships, highlighting the rapid turnover of centromere-associated sequences. In conclusion, our results suggest that *Cucurbita* genomes maintain karyotypic structural stasis during post-polyploid diploidization, with karyotype evolution instead being driven by rDNA repositioning and centromere turnover events, which constitute the cytogenetic basis for species divergence in *Cucurbita*. This finding highlights the more refined cytological evolutionary mechanisms underlying karyotypic stasis, providing new insights into post-polyploid karyotype evolution.

## Introduction

Polyploidization or whole-genome duplication (WGD) is considered to be the drive force of plant species diversification and plays an important role in plant genome evolution [1–3]. During post-polyploid diploidization, parental chromosomes were typically reshuffled to revert the polyploid genome to one functionally diploid-like, which frequently results in karyotype variation involving numerical and structural changes among post-polyploid offspring [4]. This mechanism of karyotypic reshuffling has attracted significant attention, as it has been observed in many polyploids and paleo-polyploids [5, 6]. In contrast, karyotypic stasis represents an alternative cytogenetic evolutionary mechanism documented in an increasing number of polyploid and paleo-polyploids plants [7–10]. These polyploids exhibit remarkable conservation of macrostructural karyotypes, achieving diploidization and species diversity without post-polyploid karyotypic reshuffling. However, the cytological mechanisms underlying this phenomenon and how they promote speciation and diversification remain poorly understood.

Precise comparative karyotyping serves as a powerful tool for deciphering the karyotype evolutionary dynamics of polyploids. Chromosome painting based on fluorescence *in situ* hybridization (FISH) has successfully unraveled karyotype evolutionary events underlying numerous polyploids and paleo-polyploids within the Brassicaceae family [8, 11, 12]. However, chromosome-specific karyotyping of most non-model plants, including polyploids and paleo-polyploids, remains a daunting challenge, especially for those with complex genomic origins, high chromosome numbers, and small chromosome sizes. The development of flexible oligonucleotide-FISH (oligo-FISH) methods based on plant genomic data has facilitated the identification of individual chromosomes in non-model plants with complex karyotypes [13]. This approach enables detailed karyotype characterization, providing valuable tools for deciphering the origin and karyotypic evolution of related taxa, such as *Citrus*, *Populus*, *Saccharum*, *Cucumis*, and *Triticeae* [9, 14–17]. However, this approach still requires high-density oligo probes for clear signal detection, resulting in substantial synthesis costs. Recently, we successfully developed an enhanced oligo-painting (EOP) technology in cucumber, which significantly amplifies signals from ultra-low density oligo libraries, allowing high-resolution distinguishing of homologous chromosomes, which is inaccessible to the traditional oligo-FISH [18].

The *Cucurbita* genus (squashes, pumpkins, and gourds), the most genetically diverse group within the Cucurbitaceae family, includes five domesticated and more than ten wild species [19, 20]. *Cucurbita maxima*, *Cucurbita moschata*, and *Cucurbita pepo* are the three worldwide important corps not only for production but also used as rootstocks for other important cucurbit crops, such as watermelon, cucumber, and melon [21, 22]. *Cucurbita* species have an average genome size of ~350 Mb (2*n* = 40), like other cucurbit crops, watermelon, cucumber, and melon (2*n* = 14–24) [23], but have almost twice the number of chromosomes. Genomic data demonstrate that *Cucurbita* underwent an ancient polyploidization event and retained both ancestral chromosome structures during post-polyploid diploidization [19, 24, 25]. The successive sister genus *Sicana* shares the same chromosome number (2*n* = 40) and a WGD event [26]. However, it remains unclear whether this unusual karyotypic stability persists across related species, and understanding of the cytological evolutionary mechanisms underlying karyotype diversity during post-polyploid diploidization remains limited.

Here, we developed a comprehensive library of chromosome-specific oligo probes using genomic data from cultivated *Cucurbita* species, including *C. moschata, C. moschata*, *C. pepo*, and *Cucurbita argyrosperma*. We achieved efficient signal amplification from low-density oligo probes by integrating EOP technology with optimized oligo redesign strategies for enhancer recruitment. This is a breakthrough that enabled reliable and accurate identification of all individual chromosomes across *Cucurbita* species (Fig. S1). Leveraging chromosome-specific probes combined with rDNA and centromeric markers, we conducted comparative chromosome painting analyses across six *Cucurbita* species and one sister outgroup species (*Sicana odorifera*). Our studies demonstrate extremely conserved chromosomal synteny across all 20 chromosomes among *Cucurbita* species, reflecting a remarkable stasis of karyotype structure. In addition, we also observed the subtle karyotypic differentiation among *Cucurbita* species, driven by rDNA repositioning and centromere turnover events, constituting the cytogenetic basis for species divergence in *Cucurbita*. These results highlight that post-polyploid karyotypic stasis can evolve through localized, specific genomic changes rather than requiring chromosome reshuffling, providing a new explanatory framework for post-polyploid karyotype evolution.

## Results

### Development of chromosome-specific enhanced oligo-painting in *Cucurbita*

**Figure 1:**
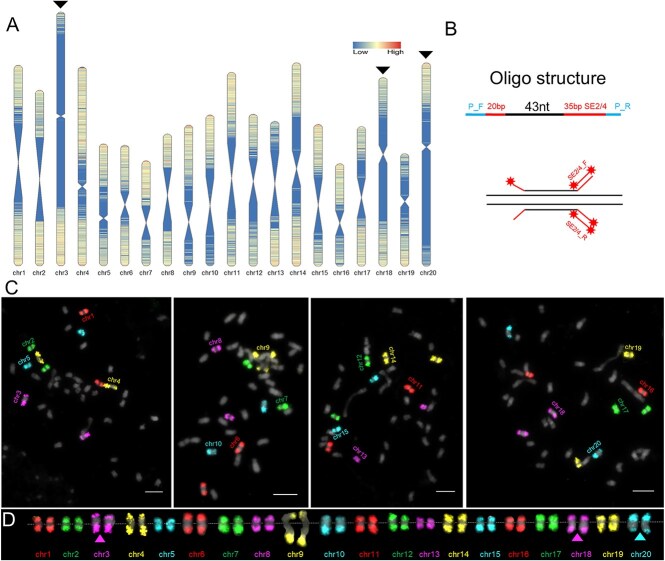
Development of chromosome-specific enhanced oligo-painting based on the *Cucurbita maxima* genome. (A) The 20 chromosomes of *C. maxima* were divided into 100 kb windows, and the number of conserved oligos was calculated for each window. The distribution of oligo density along the chromosome is shown in the heat map. Three arrows indicate three chromosomes with giant oligo-gaps. (B) The structural schematic of each oligo, based on the enhanced oligo-painting design, comprises a 43-nt genomic sequence, 20 bp signal-enhancer (SE1/2) and 35 bp SE2/4 sequences, and the sub-library-specific P-primers flanking the outermost sides. The 35 bp SE sequences were utilized in FISH experiments to recruit fluorescence-labeled secondary enhancers (SE2/4_F indicates fluorescence-labeled secondary enhancer, and SE2/4_R indicates the complementary fluorescence-labeled secondary enhancer), thereby achieving signal amplification. (C) The 20 chromosome-specific probes were performed enhanced oligo-painting (EOP) on four metaphase cells for chromosomal identification and coloring with five pseudocolors. (D) Each pair of homologous chromosomes from Fig. C was digitally excised and arranged in the order of short arm-centromere-long arm, with white dashed lines indicating centromere positions. Three arrows mark the observed signal gaps corresponding to the three chromosomes highlighted in Fig. A. Bars = 5 μm.

To identify the 20 chromosomes in the *Cucurbita* genus, the genome-wide 43-nucleotide (nt) oligos were designed using the telomere-to-telomere (T2T) genome of *C. maxima* (HZAU) [24]. These oligos were aligned to other *Cucurbita* genomes, including *C. moschata*, *C. pepo*, and *C. argyrosperma* [23], and only those with successful mappings were retained. A total of 570 871 conserved oligos were identified across the four *Cucurbita genomes* (Fig. S2). These oligos are not uniformly distributed along the chromosomes but are predominantly concentrated within approximately 6 Mb regions proximal to the chromosome ends (Fig. 1A). The terminal regions of the short arms of chr3, chr9, and chr10, as well as the long arm of chr20, exhibited relatively low oligo density, likely due to the abundance of repetitive sequences in these regions. Near-centromeric regions rich in repetitive sequences displayed gaps in oligo coverage. The proportions of these oligo gaps in chromosome length varied among chromosomes, with over 50% observed in chr3 (67.3%), chr18 (52.4%), and chr20 (57.3%) (Fig. 1A, arrows).

Four oligos were randomly selected every 10 kb from the conserved oligo pool to optimize library synthesis costs, resulting in a final set of 59 664 oligos for chromosome-specific library construction. The average oligo density across the 20 chromosomes ranged from 0.1 to 0.26 oligos/kb (Table S1). Using the enhanced oligo-painting (EOP) method [18], each oligo was redesigned to include primers (P_F/R) and signal-enhancer sequences (SE). Specifically, each chromosome-specific oligo was flanked by primer P-F, a 20-bp SE, a 35-bp SE, and primer P-R (Table S1). The P primers facilitated PCR amplification of sub-libraries from the total oligo library. Fluorescently labeled SEs generated double-stranded probe pools from the sub-libraries by PCR. The 35-bp SEs will recruit signal-enhancers to amplify fluorescent signals (Fig. 1B).

### Chromosome identification of *C. maxima based on EOP*

The odd- and even-numbered chromosomes were labeled with TAMR (red) and FAM (green), respectively. These 20 painting probes (chr1-chr20) correspond to pseudochromosomes chr1 to chr20 of *C. maxima*. These probes were hybridized on metaphase chromosomes of *C. maxima* using EOP technology. The 20 chromosomes were divided into four groups with five consecutive chromosomes each. Three rounds of EOP were performed to independently identify each set of five chromosomes in a single metaphase cell, assigning them five distinct pseudocolors. The 20 chromosomes were then visually identified across four metaphase cells (Fig. 1C). Each probe pool generated two bright and specific signals on the homologous chromosomes, confirming that these probes can be used to identify all *Cucurbita* chromosomes. Signal gaps were primarily observed in the pericentromeric regions. These results further demonstrate the effectiveness of EOP in enhancing painting signals while significantly reducing the high oligo design density typically required for high-quality oligo painting.

The colored 20 chromosomes were digitally excised from Fig. 1C and arranged according to the short arm-centromere-long arm (Fig. 1D). The painting results showed that the pericentromeric regions exhibited significant variability in the size of signal gaps among chromosomes. Relatively large signal gaps were observed on five chromosomes (chr3, chr6, chr9, chr18, and chr20). Specifically, the signal gaps on chr3, chr18, and chr20 accounted for approximately 35.8%, 38.9%, and 41.9% of the total lengths of their chromosomes, respectively (Fig. 1D, arrows), which significantly deviated from the theoretical proportions of oligo gaps. The oligo gaps on chr6 and chr9 both accounted for approximately 21% of their chromosome lengths. The observed signal gaps in metaphase chromosomes were approximately 32.1% and 53.7%, respectively, indicating that the degree of heterochromatin condensation varies significantly across chromosomes. Furthermore, these results highlight the necessity of karyotype visualization based on metaphase cells in genome assembly studies, as it can reveal the true state of chromosomes, which may substantially differ from the predicted karyotype based on sequencing data.

### Conserved chromosomal synteny of *Cucurbita species revealed by* cross-species EOP

**Figure 2:**
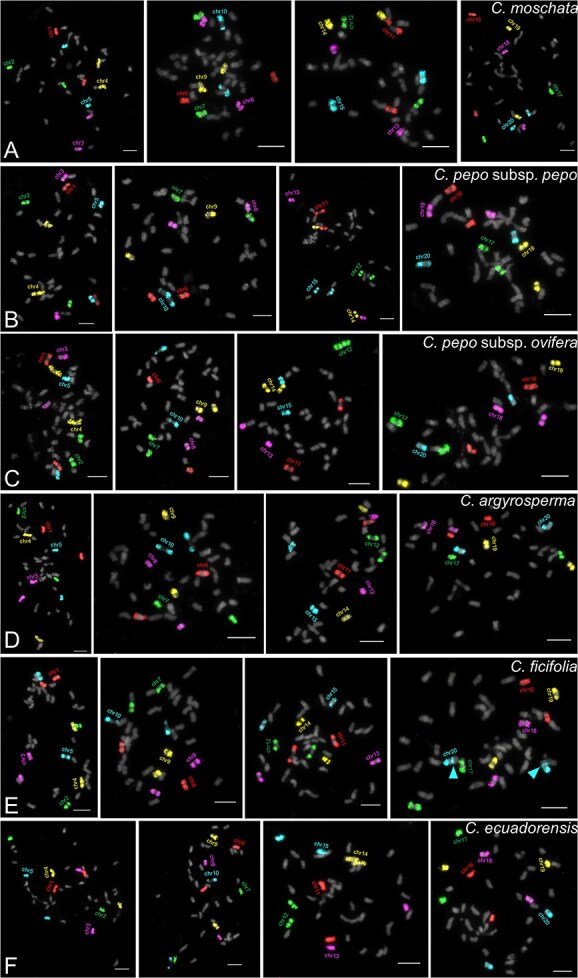
Cross-species enhanced oligo-painting based on 20 chromosome-specific probes in five other *Cucurbita* species. The 20 chromosome-specific probes were hybridized on four metaphase cells from *C. moschata* (A), *C. pepo* subsp. *pepo* (B), *C. pepo* subsp. *ovifera* (C), *C. argyrosperma* (D), *C. ficifolia* (E), and *C. ecuadorensis* (F), respectively. (E) Arrows indicate detected inversion events located on the chr20 long arm. Bars = 5 μm.

To characterize the karyotypes of different clades of *Cucurbita* species, comparative karyotyping was conducted in six *Cucurbita* species: the five domesticated crops and two phylogenetically proximate wild species (Fig. S1) [20, 26]. The 20 chromosome-specific painting probes, derived from the *C. moschata* reference genome, were utilized for cross-species EOP in the other five species. Sequential EOP experiments across four metaphase cells per species enabled the precise identification of individual chromosomes (Fig. 2). The EOP results showed that all probes generated distinct and bright painting signals in the other six tested species (Fig. 2). Notably, each species retained exactly two homologous copies per chromosome, demonstrating unequivocal one-to-one chromosomal synteny across these species. No marked interchromosomal translocations were detected, indicating highly conserved related DNA sequences among these species. This conserved chromosomal synteny implies minimal gross chromosome reshuffling during ~10 millennia of post-domestication divergence.

Additionally, *Cucurbita ficifolia* exhibited a unique chr20 short-arm signal pattern: proximal long-arm hybridization signals localized adjacent to the centromere, contrasting with the distal long-arm positioning seen in all other species (Fig. 2E, arrows). This chromosomal divergence provides direct cytogenetic evidence for *C. ficifolia*’s unique domestication trajectory, likely driven by a lineage-specific inversion event.

### Paleo-allotetraploid origin of *Cucurbita* confirmed by karyotype characteristics of *S. odorifera*

**Figure 3:**
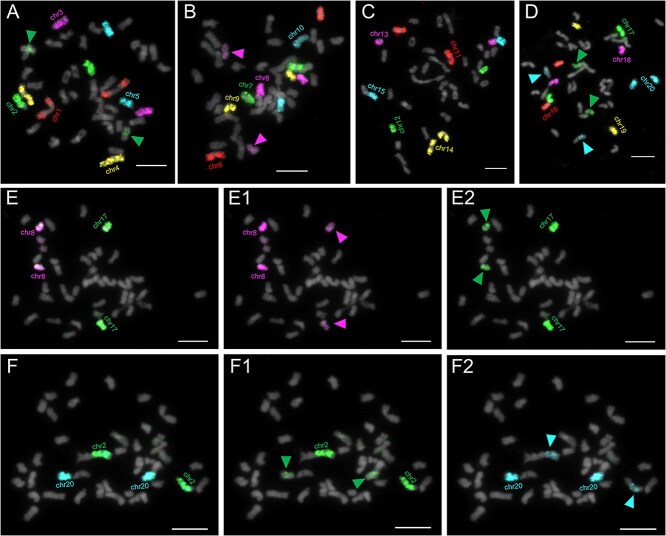
Cross-species enhanced oligo-painting based on 20 chromosome-specific probes in *Sicana odorifera*. (A–D) The 20 chromosome-specific probes were hybridized on four metaphase cells from *S. odorifera* through four rounds of FISH experiments. Arrows indicate detection of additional painting signals from chr2, chr8, chr17, chr20 probes. (E) Chr8 and Chr17, as homoeologous chromosomes, exhibited four overlapping signals—two strong and two weak (E1 and E2). (F) Chr2 and Chr20, as homoeologous chromosomes, exhibited four partially overlapping signals—two strong and two weak (F1 and F2). Two weak chr20-signals are located only in the middle region of the chr2. Bars = 5 μm.

*Sicana odorifera* is the successive sister group to *Cucurbita*, sharing the same chromosome number (2*n* = 40) and a WGD event with *Cucurbita* [11]. However, the chromosomal evolutionary relationship between the two genera has been overlooked. Therefore, we were intrigued by the chromosomal composition of *S. odorifera* compared to that of *Cucurbita* species. We further investigated whether *S. odorifera* maintains chromosomal synteny with *Cucurbita* species. Sequential cross-species EOP were performed using the same set of chromosome-specific painting probes on the metaphase chromosomes of *S. odorifera* (Fig. 3A–D).

The EOP results showed that all probes generated distinct and intense painting signals on metaphase chromosomes, further corroborating the close phylogenetic relationship between *S. odorifera* and *Cucurbita* species. The 16 painting probes consistently detected two copies of painting signals, indicating a precise one-to-one correspondence between these 16 chromosomes and those of *Cucurbita* species, along with preserved chromosomal synteny. Intriguingly, however, each of the remaining four painting probes (chr2, chr8, chr17, and chr20) was hybridized on the four chromosomes, with two chromosomes displaying strong signals and the other two exhibiting weak signals (Fig. 3A, B, and  D, arrows). This illustration suggested that the chromosomes with strong signals corresponded to chr2, chr8, chr17, and chr20, while the weak-signal chromosomes were identified as homoeologous. Further characterization of these chromosomes using painting probes established homoeologous relationships between chr8 and chr17, as well as between chr2 and chr20 (Fig. 3E and F). This finding suggested that *S. odorifera* occupies an evolutionary position closer to the ancestral lineage of *Cucurbita* species owing to its incomplete loss of homology between ancestral subgenomes. These observations provide compelling cytogenetic evidence supporting the paleo-allotetraploid origin of *Cucurbita* species, while demonstrating differential rates of homologous loss between ancestral genomes along chromosomes during the post-polyploid diploidization.

### Karyotypic diversification of *Cucurbita* accompanied by frequent rDNA repositioning

**Figure 4:**
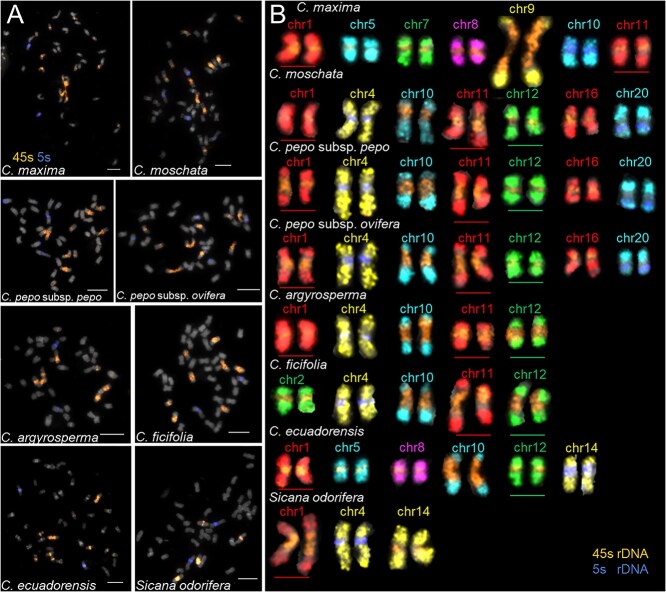
Characterization of 45S and 5S rDNA in seven *Cucurbita* species. (A) The fluorescence in situ hybridization (FISH) mapping of 45S and 5S rDNA probes on metaphase chromosomes of six *Cucurbita* species and one related outgroup species (*Sicana odorifera*). (B) Chromosomes carrying rDNA in each species were identified based on 20 oligo probes. FISH signals from rDNA and chromosome-specific oligos were merged. These colored chromosomes were digitally excised and systematically arranged by species for comparative analysis. The horizontal lines indicate the presence of 45S rDNA size differences on the corresponding chromosomes. Bars = 5 μm.

The chromosomal distribution of ribosomal DNA (rDNA) loci provides critical phylogenetic signatures for reconstructing species relationships and quantifying evolutionary divergence. 45S and 5S rDNA have been widely adopted as cytogenetic markers for karyotyping [15, 27], but the rDNA characteristics of *Cucurbita* remain cytogenetically underexplored. Therefore, we conducted comparative rDNA mapping across seven species using FISH with locus-specific oligo-probes (Fig. 4A). The 45S rDNA displayed striking interspecific variation: four signals in *S. odorifera*, compared to eight in *C. argyrosperma* and *C. ficifolia*, ten in *Cucurbita ecuadorensis*, *C. pepo*, and *C. moschata*, with an exceptional twelve signals uniquely observed in *C. moschata*, all pericentromerically anchored. For 5S rDNA, both *C. moschata* and *C. pepo* exhibited four signals (pericentromeric and terminal short-arm positions), contrasting with only two conserved pericentromeric signals in other species. This dynamic rDNA numerical variation across *Cucurbita* species highlights their lineage-specific evolutionary plasticity.

We precisely characterized rDNA-bearing chromosomes using chromosome-specific EOP (Fig. 4B). The conserved 45S/5S rDNA localization patterns between *C. pepo* and *C. moschata,* 45S signals on chr1, chr10, chr11, chr12, chr16 and 5S on chr4, 20 (Fig. 4B), providing strong phylogenetic evidence for their common evolutionary origin. *Cucurbita argyrosperma* lacked detectable 45S and 5S signals on chr16 and chr20, while *C. ficifolia* uniquely acquired a 45S locus on chr2 and lost on chr1. *Cucurbita maxima* exhibited extensive rDNA redistribution (45S: chr1, chr5, chr7, chr8, chr9, chr11; 5S: chr10), whereas *C. ecuadorensis* retained a different distribution (45S: chr1, chr5, chr8, chr10, chr12; 5S: chr14), supporting ancestral divergence followed by independent domestication trajectories. *Sicana odorifera* distinguished itself through 45S restriction to chr1 and chr14 while maintaining conserved 5S positioning. Therefore, frequent rDNA repositioning events, accompanied by the programmed elimination of specific rDNA loci, have occurred during the evolutionary trajectories of these species.

Given that hybridization intensity served as a reliable indicator of rDNA copy number, signal size showed homoeolog-specific 45S rDNA copy number dynamics (Fig. 4B, underlined). For chr1, 45S signals progressively diminished in strength across *S. odorifera*, *C. pepo*, *C. ecuadorensis*, *C. maxima*, *C. argyrosperma*, and *C. moschata*. Similarly, the size of 45S rDNA on chr11 and chr12 exhibited marked interspecific differences. These results suggest that the copy number of 45S rDNA experienced either amplification or elimination during the evolution of *Cucurbita* species. An overview, these observations demonstrate rDNA repositioning and duplication/deletion events underlying *Cucurbita* karyotype evolution, reflecting species-specific diploidization processes following polyploidization in plants and highlighting the pivotal role of rDNA in shaping chromosomal architecture.

### Centromere evolution of *Cucurbita* marked by dynamic sequence turnover

**Figure 5:**
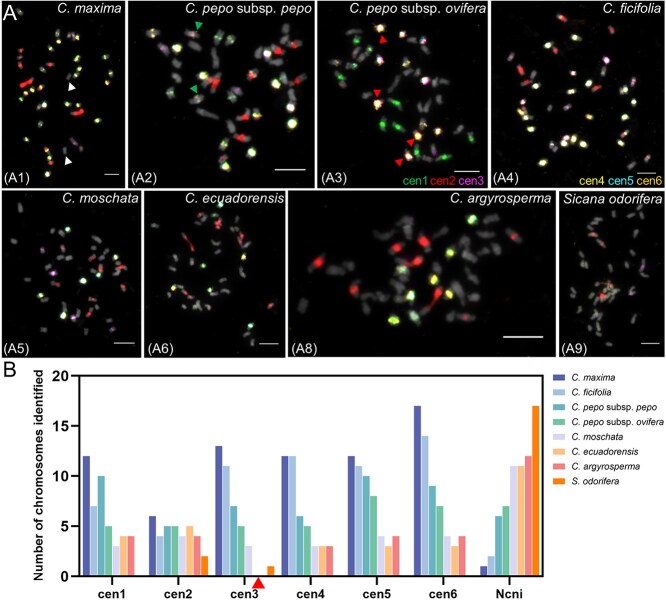
Comparative mapping of six predicted centromere monomers among seven species. (A) The six centromeric monomer probes (cen1 to cen6) were hybridized to the one metaphase cell from six *Cucurbita* and one sister outgroup species, respectively, with different pseudocolors for each monomer to facilitate visualization and analysis. *C. maxima* (A1), *C. pepo* subsp. *pepo* (A2), *C. pepo* subsp. *ovifera* (A3), *C. ficifolia* (A4), *C. moschata* (A5), *C. ecuadorensis* (A6), *C. argyrosperma* (A7), and *S. odorifera* (A8). (A1) Arrows indicate chromosomes that were not identified by the monomer probes. (A2) Arrows indicate a pair of cen1 signals deviating from the centromeric region. (A3) Arrows partially indicate that cen3 to cen6 co-localize with cen2. (B) Mapping features of six centromere monomers among seven species. Ncni indicates the number of chromosomes not identified by all monomers. Arrow points to complete loss of cen3 signal in two species (*C. ecuadorensis* and *C. argyrosperma*).

Based on the T2T genome assembly of *C. maxima*, six types of centromeric monomers were identified and designated as CEN169, CEN253, CEN315, CEN324, CEN327, and CEN654 [24]. CEN169 was localized to 12 chromosomes, CEN253 to 7 chromosomes, and CEN315 exclusively to chr4. The three monomers (CEN324, CEN327, and CEN654) were co-localized on chr2. To validate the precise localization of these monomers, we designed and synthesized ds-oligo probes of six centromeric monomers, named cen1 (CEN169), cen2 (CEN253), cen3 (CEN315), cen4 (CEN324), cen5 (CEN327), and cen6 (CEN654), respectively (Table S1). Through three rounds of FISH on metaphase chromosomes of *C. maxima*, each monomer was assigned a distinct pseudocolor (Fig. 5A). The FISH showed differences from the predicted results in that the six probes were collectively localized to 19 chromosomes (Fig. 5B and Table S2), with cen1 (12), cen2 (6), cen3 (13), cen4 (12), cen5 (12), and cen6 (17) distributing variably across centromeric domains. One chromosome pair remained unlabeled (Fig. 5 A1, arrows), suggesting the potential existence of other unidentified centromeric monomers. Notably, cen1–cen6 exhibited copy-number heterogeneity across chromosomes, as evidenced by changes in signal intensity. This observation further underscores the complexity and diversity of centromere evolution in *Cucurbita* species. However, striking discordance between sequence-predicted and experimentally validated monomer distributions highlights the inherent limitations of centromere annotation from T2T assemblies, emphasizing the indispensability of cytological validation.

We performed FISH using these monomeric probes across the other six species studied (Fig. 5A2–A8 and Fig. S3), followed by quantification of the identified chromosomes (Fig. 5B and Table S2). The results revealed a phylogenetically correlated decline in the number of chromosomes harboring these centromeric monomers across these species, mirroring their evolutionary divergence in centromere architecture. Beyond interspecific monomer-localization heterogeneity and copy-number polymorphisms, we observed intraspecific differentiation in *C. pepo* subspecies: subsp. *pepo* exhibited two cen1 locus dissociation from canonical centromeric regions (Fig. 5A2, arrows), while subsp. *ovifera* uniquely featured cen3–cen6 co-localization with cen2 but not cen1, a configuration distinct from all other species (Fig. 5A3 and Fig. S3C, arrows). These findings underscore the rapid repositioning of these centromeric monomers during subspecies radiation.

Cytological mapping revealed complete spatial overlap between cen2 and 45S rDNA loci (Fig. S4), demonstrating the co-evolution of 45S with centromeres. Cen3 displayed taxonomic depletion, showing no detectable signals in *C. ecuadorensis* and *C. argyrosperma* (Fig. 5B and Fig. S3), indicating its specific elimination within these two species. The cen4, cen5, and cen6 displayed clade-dependent co-localization patterns (Fig. S3). Remarkably, *S. odorifera* exhibited near-total depletion of predicted centromeric monomers, only a few signal traces of cen2 and cen3 were observed (Fig. 5A8 and Fig. S3), signifying near-complete turnover of ancestral centromeric elements. In summary, our data illuminated a dynamic evolutionary landscape of centromeres in *Cucurbita* with high diversity and extensive remodeling, characterized by dynamic sequence turnover: large-scale elimination and de novo emergence of centromere-associated satellite DNA at both inter- and intraspecific levels.

### Karyotypic structural stasis of Cucurbita species based on comparative karyotyping

**Figure 6:**
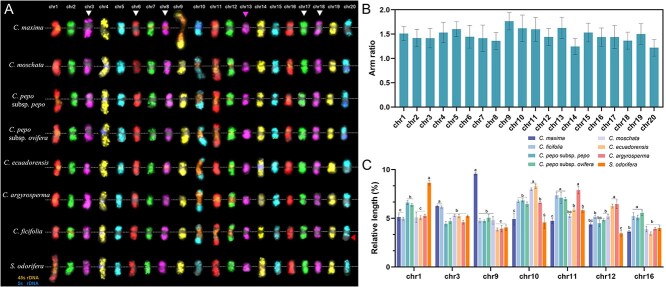
Comparative karyotyping of seven *Cucurbita* species integrating single-copy oligo and rDNA-painting. (A) Well-spread metaphase cells were selected for sequential FISH experiments using chromosome-specific and rDNA oligo probes to identify all chromosomes. The colored chromosomes of each species were digitally excised and systematically arranged for comparative karyotype analysis. Arrows indicate variations in signal gaps across seven species. Dashed lines denote cytological centromere positions. (B) Schematic representation of arm ratio ranges among all seven species. (C) Comparative analysis of relative chromosome lengths of seven morphologically different chromosomes. Error bars represent standard deviation (SD, n = 10). Lowercase letters indicate significant differences at *P* < 0.05 (ANOVA, Turkey’s correction).

Chromosome-specific painting probes derived from *C. maxima* enabled precise chromosomal identification across selected species. We established comparative karyotypes for these species based on the unambiguous recognition of individual chromosomes (Fig. 6A). Despite conserved synteny across seven species, some significant karyotypic variability was observed, mainly regarding rDNA features, cytological centromere sizes, and relative lengths. Besides the cross-species variation in rDNA loci, we also visualized changes in signal gap size on chr3, chr6, chr8, chr17, and chr18 (Fig. 6A, arrows), suggesting species-specific expansions or contractions of repetitive sequences in these centromere regions. We systematically measured 10 well-spread metaphase cells from each species to quantify each chromosome morphology (Table S3). All chromosomes across the seven species exhibited submetacentric morphology with a mean arm ratio of approximately 1.5 (long arm length/short arm length) (Fig. 6B) [28, 29], showing no significant interspecific variation even for chromosomes involved in rDNA repositioning (Fig. S5). These findings indicated that chromosomes remained structural stasis during *Cucurbita* speciation, implying that frequent rDNA repositioning at centromeres did not induce substantial alterations in chromosome type.

We further found that the relative length of several chromosomes showed significant variation across seven species (Fig. S6 and Table S3). The 10 chromosomes (chr2, chr4, chr6, chr7, chr8, chr15, chr17, chr18, chr19, and chr20) were identified with conserved relative lengths across species without significant differences. Notably, chr13, the shortest chromosome, displayed a prominent centromeric signal gap in *C. ficifolia* (Fig. 6A). Similar patterns were observed in chr3, chr6, chr8, chr17, and chr18 (Fig. 6A, white arrows). Additionally, chr5 in *C. ficifolia* exhibited an expanded gap, resulting in significantly greater relative length compared to homologs in other species. These observations suggested that the accumulation of centromere-associated repetitive DNA leads to the dramatic size variations of centromeres among species, potentially associated with rapid centromere turnover.

Among seven chromosomes showing interspecific length variations (Fig. 6C), chr1, chr10, chr11, chr12, and chr16 displayed length changes correlated with 45S rDNA amplification or depletion (Fig. 6A). Chr3 length variations were closely related to the differential percentage of repeat regions associated with the signal gaps. Strikingly, chr9 in *C. maxima* became the largest chromosome due to massive 45S rDNA amplification at its centromeres. These results demonstrated that dynamic 45S rDNA alterations drive major chromosomal length polymorphisms without affecting centromere positioning, whereas 5S rDNA alterations exert negligible effects on chromosome morphology. In conclusion, while *Cucurbita* species maintain karyotypic structural stasis during diploidization, their karyotypes have undergone fine-scale differentiation through rDNA- and centromere-mediated dynamics, potentially raising hybridization barriers between *Cucurbita* species.

## Discussion

While comparative karyotyping remains the gold standard for investigating chromosomal evolution among closely related species, the precision of such comparison hinges on accurate chromosome identification, a persistent challenge in non-model plants and plants with small chromosomes. Recent advances in oligo-FISH technology have revolutionized this situation, enabling high-resolution comparative karyotyping in genetically complex plant systems, and have been widely used in several plant taxa [9, 14, 15, 17, 29–32]. However, this oligo-FISH still requires a high density of oligos, especially unfriendly to large genomes requiring higher library synthesis costs. The EOP we developed breaks chromosome-scale limitation and can be widely applied in various genome-scale plants. This cutting-edge approach in plants offers three key advantages: customizable probe design for species-specific applications, superior signal-to-noise ratio through optimized oligo library engineering, and cost-effective comparative painting for homologous/homoeologous.

The *Cucurbita* genus presents particular cytogenetic challenges compared to the well-characterized *Cucumis* genus due to its high chromosome number, small size, high similarity, and lack of reliable chromosomal markers. Here, we developed a novel set of EOP probes using conserved sequences from the T2T-assembled genome of *C. maxima*. Through optimized oligo library engineering, we achieved high-fidelity and cost-effective chromosome-specific identification with ideal resolution. These EOP probes have demonstrated high reliability across *Cucurbita* species, enabling accurate comparative karyotyping of these species.

We use these probes to describe, for the first time, comparative karyotyping across six *Cucurbita* species and one related outgroup (*S. odorifera*). Intriguingly, we detected homoeologous signals between chr8-chr17 and chr2-chr20 in *S. odorifera*, a pattern absent in *Cucurbita* species. This finding provides direct cytological evidence for their shared allotetraploid origin, likely involving hybridization between two divergent diploid progenitors followed by polyploidization [19, 25, 26]. Extensive chromosome reshuffling is common in many allopolyploids, such as *Brassica napus*, *Saccharum, Tragopogon miscellus*, and crucifer tribe [5, 15, 33, 34]. However, the extremely conserved chromosome synteny in *Cucurbita* suggests no chromosome reshuffling but rather karyotypic stasis following polyploidization. This ‘freezing’ (maintaining subgenomic stability) has been found in an increasing number of polyploid plants, such as *Camelina*, *Gossypium*, *Eragrostis*, and London planetree [10, 12, 35, 36], demonstrating that nonhomeologous chromosome shuffling s may not always happen following polyploidization.

Early-stage homology sequence elimination and neo-functionalization in polyploids facilitate diploidization by addressing meiotic chromosomal mispairing, a primary driver of karyotypic reshuffling in offspring. As observed in *S. odorifera* and *Cucurbita*, partial or complete elimination of homology sequences suggests that their genomes appear to achieve diploidization primarily through selective sequence elimination rather than large-scale chromosomal reshuffling. This explains why *Cucurbita* genomes comparable in genome size to diploid cucurbits like cucumber, melon, and watermelon, maintain a constant chromosome number (2*n* = 40). The innate preference of homologous chromosome pairing favors partners with the most similar DNA sequences and can be discriminated based on cryptic sequence variation in nascent allopolyploids [37]. As sequence elimination increases divergence between parental genomes or parental genomes are sufficiently divergent, chromosomal mispairing becomes less frequent or negligible, then genome diploidization involving chromosome reshuffling should be less essential and have a slower pace [8]. This seems to be true for the allotetraploid genome of *Cucurbita* and can explain (at least partially) the absence of extensive chromosome reshuffling but rather karyotypic stasis after polyploidy in *Cucurbita*.

The rDNA, including 45S and 5S, has long served as a fundamental cytogenetic marker for studying karyotype characteristics, providing essential insights into species phylogeny and chromosome evolution mechanisms. In this study, we systematically characterized the rDNA features of seven species using rDNA in combination with chromosome-specific probes. The results revealed that, despite these species maintaining robust karyotypic stasis, their rDNA locus distribution exhibited remarkable diversity, closely associated with the diversification and speciation of *Cucurbita* species. The number of rDNA loci tends to increase with ploidy level rather than chromosome number, reflecting the potential implications of WGD events [27]. However, during the diploidization process following polyploidization, the number of rDNA loci per monoploid genome showed a significant reduction, a phenomenon observed in *Artemisia* and *Nicotian*a [38, 39], indicating a rapid concerted evolutionary pattern. As observed in *S. odorifera*, the presence of one 5S rDNA locus and two 45S rDNA loci suggests a more rapid elimination of rDNA loci in *S. odorifera* compared to other *Cucurbita* species.

The rDNA variation involved various mechanisms, including chromosomal rearrangements, locus duplication/deletion, and transposon-mediated transposition events [40]. In *Cucurbita* species, rDNA diversity seems to involve the latter two mechanisms rather than chromosomal rearrangements primarily. This is evident from the observed size variations of 45S loci across species, which are mediated by locus duplication/deletion. Transposon-mediated rDNA repositioning appears to play a dominant role in *Cucurbita* species, as all 45S loci localized to centromeric regions, where centromeric domains often undergo rapid evolution due to active transposon insertions [41].

Plant centromeres play a critical role in the genome, yet they exhibit remarkable inter- and intra-specific variation in size, structure, and composition. Notably, the transposon element (TE) and centromere DNA sequence can evolve rapidly when genome stability is compromised by events such as WGD. Here, we selected six centromere-associated monomer probes from *C. maxima* and conducted a systematic comparative analysis across seven species. The results revealed significant variability in these monomers across species, with marked differences in both their position and copy number. Remarkably, in *S. odorifera*, the signal was nearly undetectable. Even within the same clade, *C. maxima* and *C. ecuadorensis* exhibited substantial divergence in their centromeric features. These findings provide robust evidence for the rapid evolution of centromeres in *Cucurbita* species. This may partly explain the observed hybridization barriers among these species [42], even in the context of conserved chromosomal collinearity.

Previous research has suggested that repeat variants can translocate between different chromosomes in the cell via gene conversion [43]. As we have shown, a unique phenomenon was observed in *C. pepo* subsp*. ovifera*: the co-localization pattern of cen2-cen6 differed significantly from that in other species. This suggests that cen2-cen6 monomers may have reposition through gene conversion, while the CENH3 protein on the original chromosome potentially formed a new functional centromere through sequence turnover. The average insertion time of LTR-RTs in the centromeric regions (0.55 mya) was significantly later than that of non-centromeric regions (1.57 mya), suggesting that rapid TE insertion occurs in the centromeres of *C. maxima* [24]. The cumulative insertion of transposable elements at centromeres may facilitate the formation of novel centromeric satellite arrays, thereby driving the rapid evolution of centromeres across species [44].

## Conclusion

In summary, we performed fine comparative karyotyping across seven related species using chromosome-specific EOP combined with rDNA- and centromere-associated probes. The EOP results confirmed the hypothesis of *Cucurbita* paleo-allotetraploid origin at the chromosomal level and demonstrated that karyotypic stasis prevailed following the polyploidization of *Cucurbita*. The rDNA and centromeres were characterized by extensive variation across species, driving subtle divergence in the morphology of the associated chromosomes among *Cucurbita* species. Our findings highlight the refined cytological mechanisms driving the karyotype evolution of polyploids through localized and specific genomic fine-tuning under karyotype stasis, advancing the knowledge of how polyploidization and subsequent diploidization contribute to speciation.

## Methods

### Plant materials and chromosome preparation

Six *Cucurbita* and one related outgroup species [20, 26], including *C. maxima*, *C. moschata*, *C. pepo* subsp. *pepo*, *C. pepo* subsp*. ovifera*, *C. argyrosperma*, *C. ecuadorensis*, *C. ficifolia*, and *S. odorifera*, were used for FISH assays in this study (Fig. S1). Chromosomes are prepared from root tips as previously described with some modifications [9, 45]. The seeds were treated in moist petri dishes at 25°C to obtain root tips. Root tips were treated with 0.002 M 8-Hydroxyquinoline for 35 min at 4°C and subsequently fixation in Carnoy’s solution. An enzymatic solution with 4% (w/v) cellulase RS (Yakult, Japan), 2% pectolyase Y-23 (w/v) (Yakult, Japan), and 2% pectinase (w/v) (Macklin, China) was used to digest the root tips for 40 min at 37°C. The digested root tips were transferred onto the slide and prepared for metaphase chromosomes as previously described [45].

### Development of chromosome-specific enhanced oligo-painting

The T2T reference genomes of *C. maxima* (HZAU) were downloaded from the National Genomics Data Center BioProject database (PRJNA1060488) [24]. Chorus2 software was used to generate single-copy 43-nt oligos with the parameters ‘-l 43 -homology 75 -step 5’ [46]. To identify conserved oligos in *Cucurbita*, we sequentially aligned the original oligos to the reference genomes of *C. moschata*, *C. pepo*, and *C. argyrosperma* using BWA ALN [19, 25, 47]. Only oligos that successfully aligned were retained at each step. Through this three-stage filtration, a total of 570 871 conserved oligos were obtained. The density of these oligos was calculated per 100 kb intervals and visualized on chromosome ideograms (Fig. S2). To optimize library synthesis costs, we further filtered the conserved oligo pool with a parameter of 4 oligos per 10 kb. This process yielded 59 664 oligos (Data S1), which were distributed across each chromosome with an average density ranging from 0.1 to 0.26 oligos per kb (Table S1).

Given the EOP technology capability for effective imaging even at low oligo densities, we proceeded to reconstruct the structure of 20 chromosome-specific oligos. Specifically, we appended 20 bp SE1_F and 35 bp SE2_R sequences to the ends of oligos on odd-numbered chromosomes, while oligos on even-numbered chromosomes were flanked with 20 bp SE3_F and 35 bp SE4_R. We ligated the identical 18 bp P_F and 18 bp P_R primers to the outermost side of oligos from two neighboring chromosomes, and so forth, for a total of 10 P_F/R primer pairs (Table S1). The 35 bp SE2 and SE4 sequences were designed to recruit fluorescence-labeled complementary secondary oligos (SE2/4_F/R) during FISH experiments (Fig. 1B), thereby enhancing the FISH signal intensity. For the six centromeric monomer sequences [24], each monomer was divided into 53 nt oligos and supplemented with monomer-specific C_F/R and sub-library-specific W_F/R primers as described by Bi *et al.* [45]. Ultimately, a comprehensive library comprising 59 708 oligos (59 664 single-copy and 44 centromere oligos), each 134 nt in length, was synthesized (Genscript Biotech, Nanjing, China).

### Synthesis of chromosome-specific and centromere probes

The comprehensive oligo library was divided into 11 sub-libraries by PCR amplification using unlabeled P_F/R and W_F/R primers as published protocol [45]. The 10 chromosome-specific sub-libraries were used as templates for PCR amplification by TAMRA- or FAM-labeled 20 bp SE1/2 and SE3/4 primers as the published protocol [18]. The 20 chromosome-specific double-stranded probes were obtained from PCR products. The probes corresponding to odd- and even -numbered chromosomes were labeled with TAMRA (red) and FAM (green), respectively. Similarly, using the monomer-specific sub-libraries as templates, PCR was performed with FAM- and TAMRA-labeled C1-C6 primers to generate monomer-specific probes. The complete 45S rDNA sequences from rice (GenBank:KM036294.1) were aligned to the *C. maxima* genome. Within the aligned regions, we selected one 60-bp sequence from each of the following ribosomal RNA components: 18S rRNA gene, internal transcribed spacer 1 (ITS1), 5.8S rRNA gene, internal transcribed spacer 2 (ITS2), and 26S rRNA gene, which collectively constitute the 45S rDNA oligo set. The same method was used to obtain one 5S oligo for 5S rDNA (KM036311.1) (Table S1). These oligos were then labeled with TAMRA or FAM at 5′ ends for subsequent FISH detection.

As the published protocol [18], for secondary oligos, 33 bp SE2/4 sequences (SE2/4_F) and their complementary sequences (SE2/4_R) were modified with TAMRA or FAM at the 5′ and 3′ ends, respectively (Tsingke Biotech, Nanjing, China). Four secondary oligo libraries were finally obtained and premixed into a 10 μM working solution.

### Enhanced oligo-painting experiments

The EOP protocol was performed as described previously with some modifications [18]. Specifically, the hybridization mixtures contained 10 μL of 100% deionized formamide, 2 μL of 20× SSC, 2 μL of ds-oligo probes, 2 μL of 33 bp secondary oligos mix, and 4 μL of 50% dextran sulfate. Denatured mixtures were covered onto prepared slides and then placed at 37°C for overnight treatment. The subsequent slide washing was performed as described previously [18]. Finally, the chromosome slides were stained with 4′,6-diamidino-2-phenylindole (DAPI) (Vector Laboratories, California, USA). FISH images were captured as separate images in different channels using a SENSYS (http://www.photometrics.com) CCD camera attached to an Olympus (http://www.olympus-global.com) BX51 microscope. The CCD camera was controlled using FISH view 5.5 software (Applied Spectral Imaging Inc., http://www.spectral-imaging.com).

The procedure for reusing slides is as follows: thoroughly rinse slides under running distilled water to remove immersion oil. Place slides in a staining rack and immerse in ddH₂O until coverslips detach completely. Perform a 5-minute wash in ddH₂O with gentle agitation. Remove excess water by careful manual shaking. The processed. Subsequently, the slides were washed once with 2× SSC buffer for 5 minutes under gentle agitation, followed by two washes with distilled water. After air-drying, the slides were ready for the next round of probing. In each round of FISH, the chromosome and probe signals were captured as separate images in distinct optical channels.

### FISH image processing and karyotyping

The images were merged and pseudocolored for each chromosome or monomer signal using Image-Pro Plus 6.0 (Media Cybernetics, Inc., Silver Springs, MD, USA). Final FISH images were processed and adjusted using Adobe Photoshop CC (Adobe, San Jose, CA, USA). For chromosome morphology analysis, ten well-spread metaphase cells were selected. ImageJ software (https://imagej.nih.gov/ij/) was employed to measure individual chromosomes, calculating the arm ratio (length of the long arm/length of the short arm), total chromosome length (length of the long arm + length of the short arm), and relative length (100 × length of the chromosome length/length of all chromosomes) (Table S3).

## Supplementary Material

Web_Material_uhaf179

## Data Availability

All data are available in the manuscript or supplementary materials.
