# Supplementary material for: Chromosome-specific oligo-painting provides insights into the cytogenetic basis of karyotypic stasis in paleo-allotetraploid Cucurbita
Source: Hortic Res. 2025 Jul 8;12(10):uhaf179. doi: 10.1093/hr/uhaf179 (PMC12537017; doi:10.1093/hr/uhaf179)
Supplement: Web_Material_uhaf179 [file web_material_uhaf179.zip › Supplemental Figure.pdf]

Supplemental Information

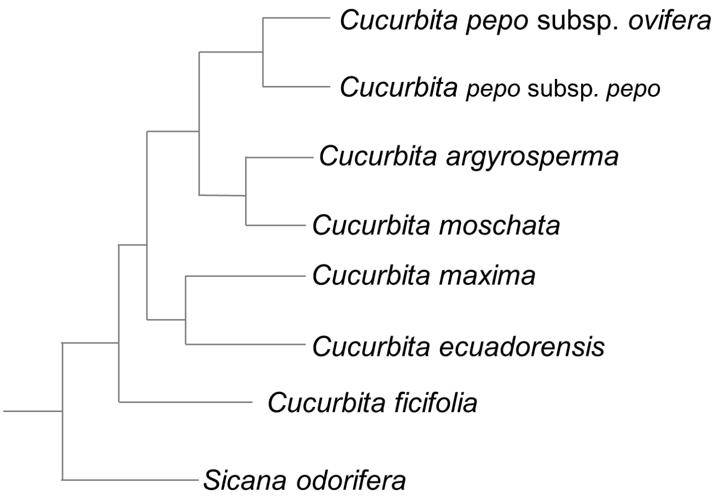

Figure S1 Phylogenetic relationships of the seven species selected for this study. Phylogenetic relationships constructed based on published data (Chomicki et al., 2020; Guo et al., 2020).

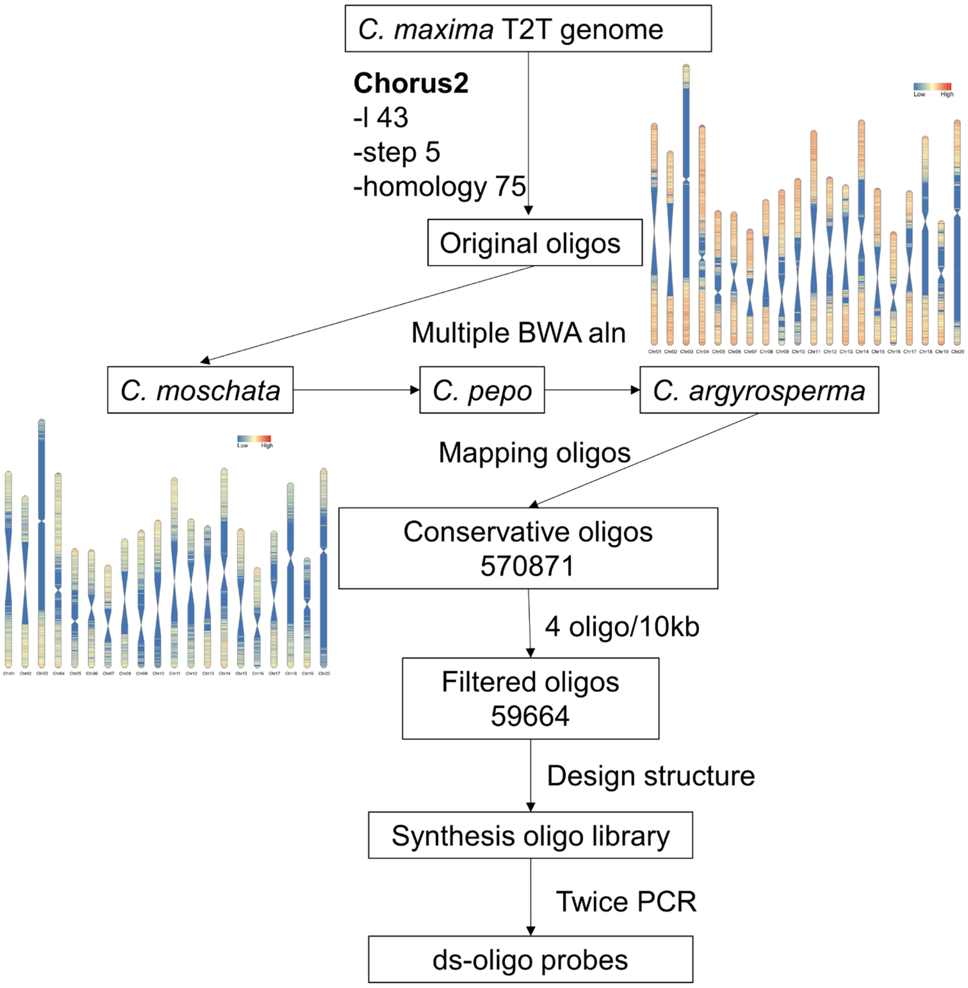

Figure S2 Development process of enhanced oligo-painting libraries.

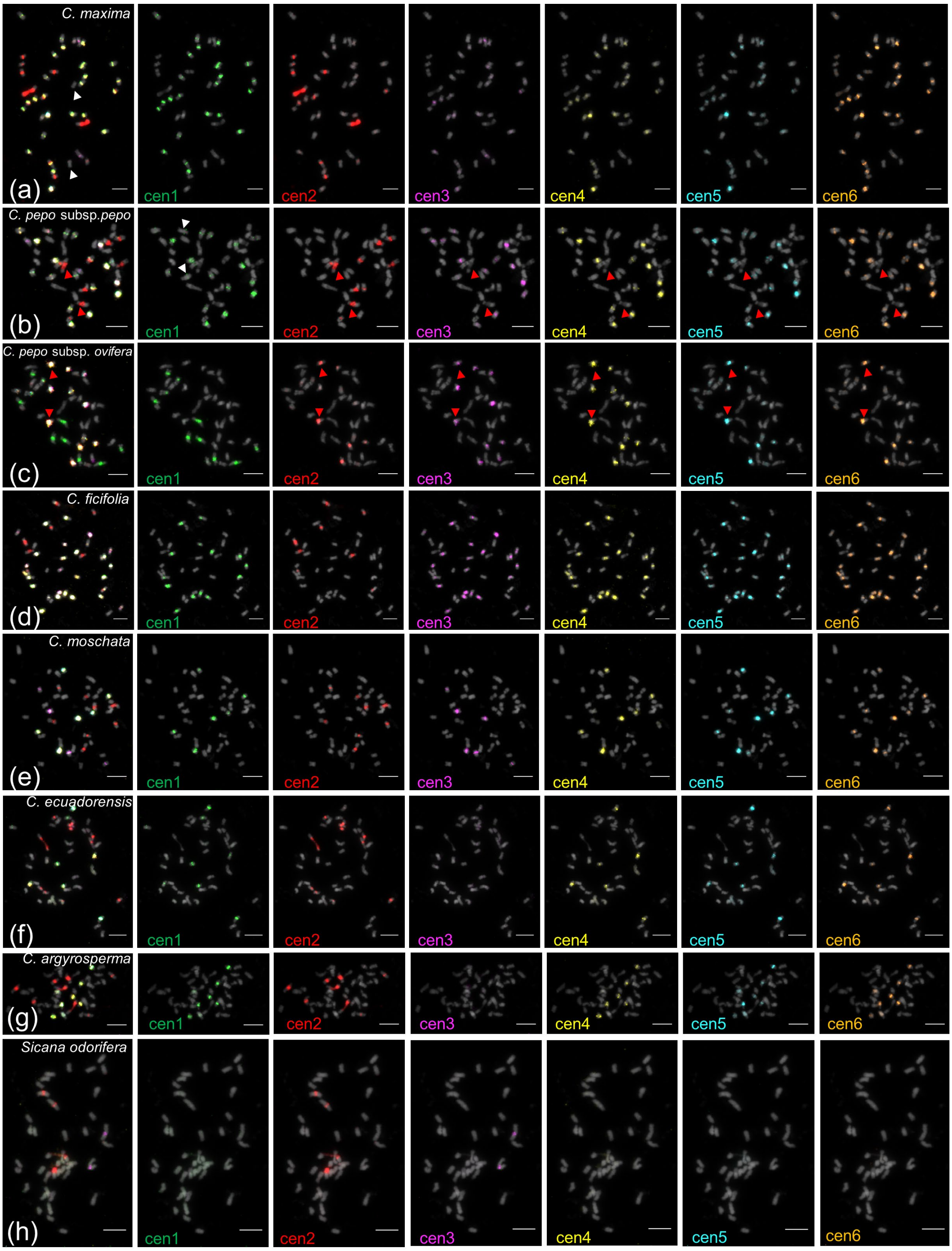

Figure S3 Mapping of six predicted centromere monomers among seven *Cucurbita* species.

The six centromeric monomer probes (cen1-cen6) were hybridized to the one metaphase cell from six *Cucurbita* and one sister outgroup species, respectively, with different pseudocolors for each monomer to facilitate visualization and analysis. *C. maxima* (a), *C. pepo* subsp. *pepo* (b), *C. pepo* subsp. *ovifera* (c), *C. ficifolia* (d), *C. moschata* (e), *C. ecuadorensis* (f), *C. argyrosperma* (g), and *S. odorifera* (h). The FISH maps of each monomer were separated and aligned.

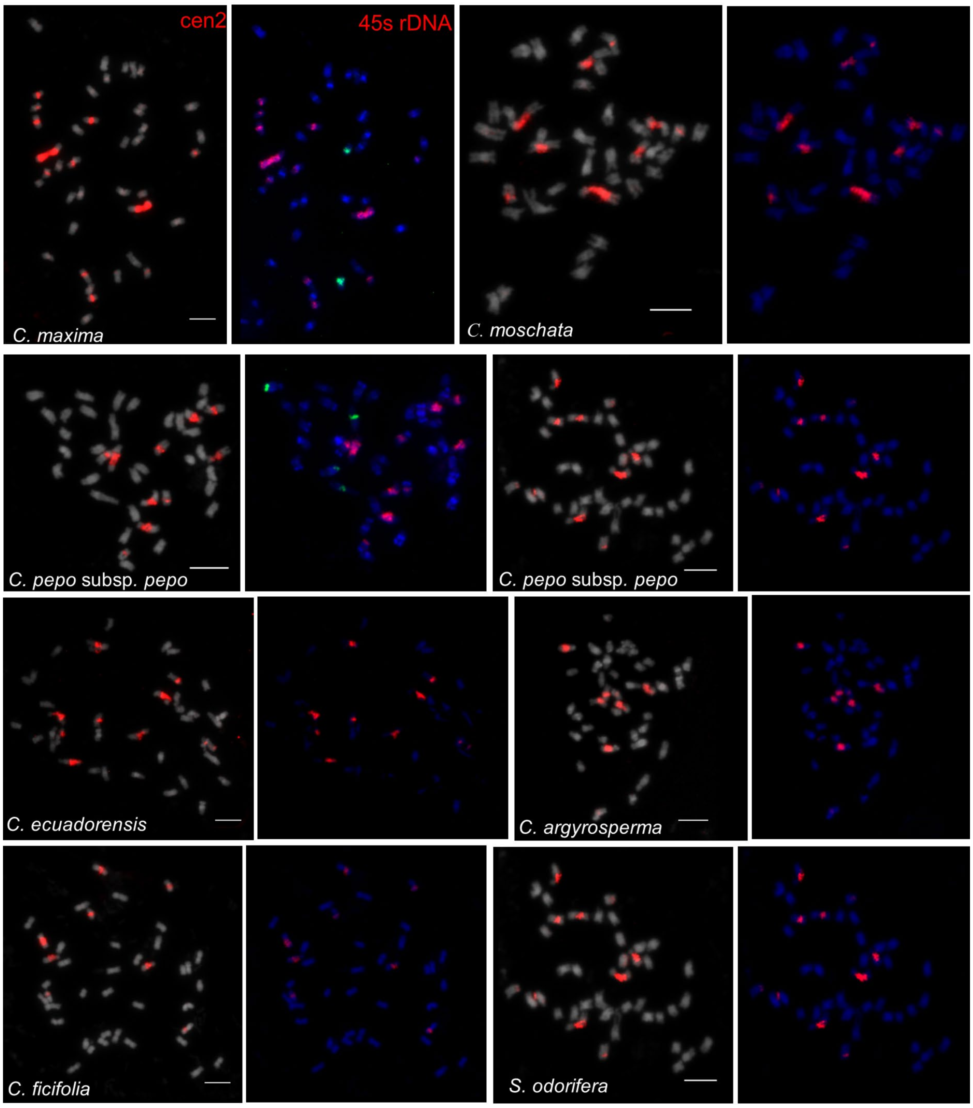

Figure S4 The cen2 and 45s rDNA co-localized in the centromere regions of seven *Cucurbita* species.

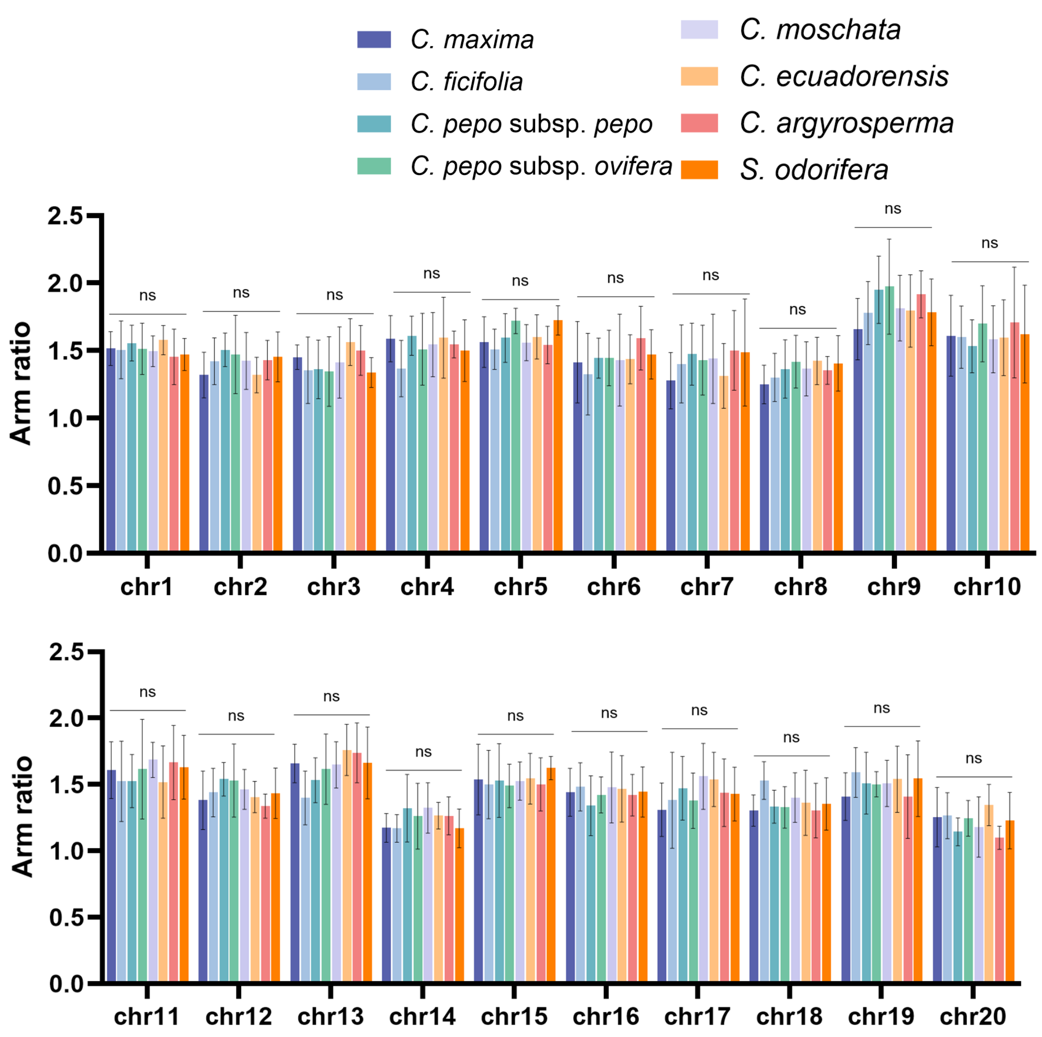

Figure S5 Comparative analysis of Arm ratios of individual chromosomes across seven *Cucurbita* species.

Error bars represent standard deviation (SD, n = 10) (ANOVA, Turkey's correction).

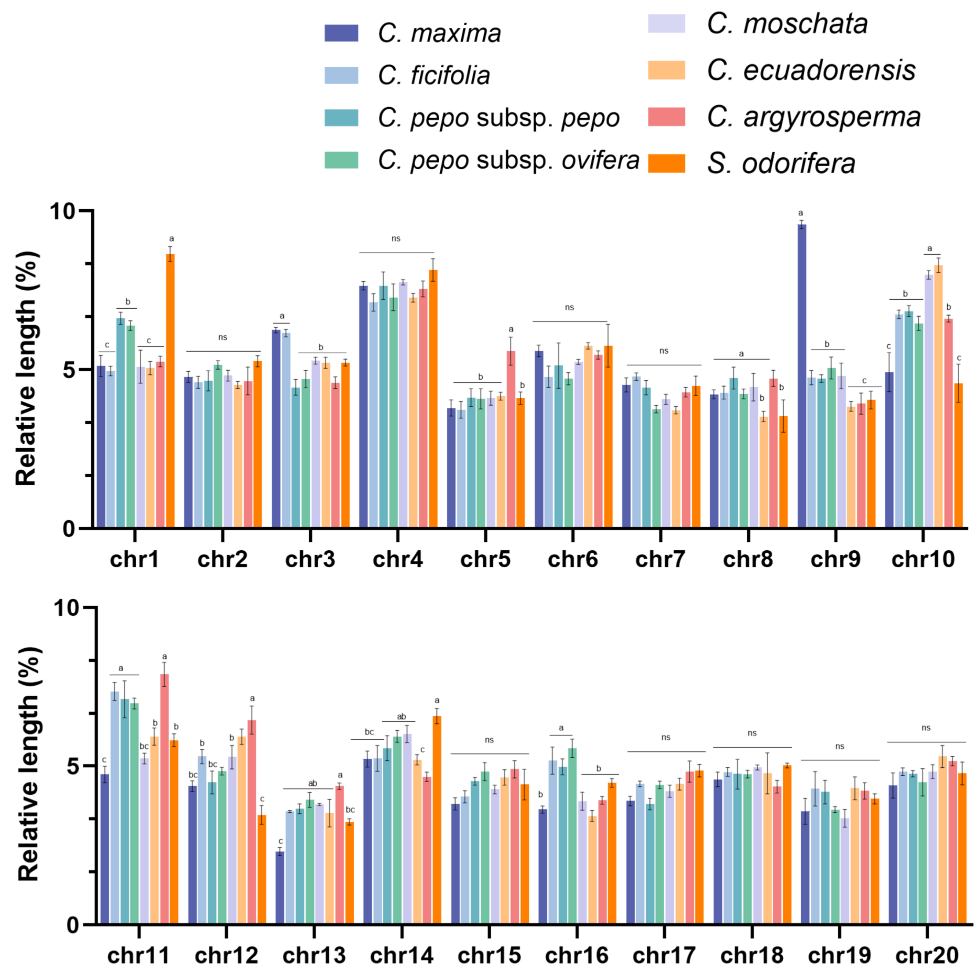

Figure S6 Comparative analysis of relative lengths of individual chromosomes across seven *Cucurbita* species.

Error bars represent standard deviation (SD, n = 10). Lowercase letters indicate significant differences at $P < 0.05$ (ANOVA, Turkey's correction).

Table S2 Mapping features of six centromere monomers among seven *Cucurbita* species.

Species	Number of chromosomes identified						Number of chromosomes not identified
	cen1	cen2	cen3	cen4	cen5	cen6	
C. maxima	12	6	13	12	12	17	1
C. ficifolia	7	4	11	12	11	14	2
C. pepo subsp. *pepo*	10	5	7	6	10	9	6
C. pepo subsp. *ovifera*	5	5	5	5	8	7	7
C. moschata	3	4	3	3	4	4	11
C. ecuadorensis	4	5	0	3	3	3	11
C. argyrosperma	4	4	0	3	4	4	12
S. odorifera	0	2	1	0	0	0	17
